# Elucidating the Pathogenesis of Pre-eclampsia Using In Vitro Models of Spiral Uterine Artery Remodelling

**DOI:** 10.1007/s11906-017-0786-2

**Published:** 2017-10-23

**Authors:** Ross McNally, Abdelrahim Alqudah, Danilo Obradovic, Lana McClements

**Affiliations:** 1 Centre for Experimental Medicine, School of Medicine, Dentistry and Biomedical Sciences, Queen’s University Belfast, Belfast, UK; 2 Institute of Pathology, University of Belgrade, Belgrade, 11,000 Serbia

**Keywords:** Pre-eclampsia, Preeclampsia, Pregnancy, Trophoblast cells, Endothelial cells, Spiral uterine artery remodelling, In vitro models, Angiogenesis

## Abstract

**Purpose of Review:**

The aim of the study is to perform a critical assessment of in vitro models of pre-eclampsia using complementary human and cell line-based studies. Molecular mechanisms involved in spiral uterine artery (SUA) remodelling and trophoblast functionality will also be discussed.

**Recent Findings:**

A number of proteins and microRNAs have been implicated as key in SUA remodelling, which could be explored as early biomarkers or therapeutic targets for prevention of pre-eclampsia.

**Summary:**

Various 2D and 3D in vitro models involving trophoblast cells, endothelial cells, immune cells and placental tissue were discussed to elucidate the pathogenesis of pre-eclampsia. Nevertheless, pre-eclampsia is a multifactorial disease, and the mechanisms involved in its pathogenesis are complex and still largely unknown. Further studies are required to provide better understanding of the key processes leading to inappropriate placental development which is the root cause of pre-eclampsia. This new knowledge could identify novel biomarkers and treatment strategies.

## Introduction

Pre-eclampsia occurs in pregnancy, and it is characterised by new onset of hypertension with proteinuria or other organ dysfunctions after 20-week gestation [1]. Pre-eclampsia is the leading cause of maternal and foetal morbidity and mortality worldwide [2]. Pre-eclampsia occurs in 4–6% of pregnancies [2]. Certain pre-existing conditions such as type 1 and type 2 diabetes mellitus (DM) can increase the risk of pre-eclampsia up to 4-fold [3, 4].

Pre-eclampsia does not only have short-term risks, but long term can lead to cardiovascular disease and/or type 2 DM in both mothers and their offspring [5, 6]. Currently, there are no reliable and early predicative biomarkers, preventative measures or treatment strategies, other than delivery. The mechanistic data related to the development of pre-eclampsia is lacking, and, as a result, the pathogenesis of pre-eclampsia is poorly understood. Some of the processes which appear to be involved in the development of pre-eclampsia include inappropriate remodelling of spiral uterine artery (SUA) likely caused by inadequate function of trophoblast cells [7]. Inadequate remodelling of SUA leads to restricted supply of oxygen and nutrients to placenta and, therefore, placental hypoxia [8].

### Spiral Uterine Artery Remodelling by Trophoblast Cells: Physiological Processes

In the early stages of embryogenesis (5 days after fertilisation), the mammalian blastula is referred to as a blastocyst, a hollow bundle of cells that has undergone minor cell differentiation. The outermost layer of the blastocyst is called the trophectoderm, which comprises of trophoblast cells. The blastocyst, following various morphogenetic events, undergoes implantation in the decidua, a membrane lining the uterus. Subsequently, trophoblast cells from the blastocyst start to migrate towards the SUA, and remodelling process begins [9]. Chorionic villi sprouting from the blastocyst are involved in invading the endometrium of the mother. This placental villous growth occurs under hypoxic conditions, aiding the proliferation of certain trophoblast cell types [10, 11]. The established oxygen gradient during placental development determines the action of the trophoblast cells, whether they migrate or proliferate [12]; this is because a change from low to high placental oxygen causes trophoblasts to develop an invasive nature, instead of proliferating [13]. Once dilation of SUA occurs by invasive trophoblasts, the change is irreversible, ensuring a constant blood flow to the developing foetus [14]. However, placental hypoxia beyond the first trimester is associated with pregnancy pathologies such as pre-eclampsia [15].

The multifaceted progression of blastocyst implantation into the decidua is governed by an array of timed mechanisms and a variety of key molecules. Human chorionic gonadotrophin (hCG) is highly expressed by the blastocyst prior to implantation [16], and hyperglycosylated hCG (hCG-H) is continually produced by the syncytiotrophoblasts subsequent to implantation [17] which, then, leads to invasion of trophoblasts [18]. Cytotrophoblasts are constantly undergoing differentiation into syncytiotrophoblasts in the floating villus to enable expansion. However, these cells also give rise to the extravillous trophoblast cells (EVTs). In relation to the villous stroma, proximal cytotrophoblast cells are differentiating, whereas distal cytotrophoblast cells are deemed column cytotrophoblasts that no longer proliferate. Hypoxic conditions have been shown to prevent the differentiation of cytotrophoblasts in vitro [10]. The differentiated syncytiotrophoblasts, which form a continuous and multinucleated syncytium, line the outer layer of the villi, whereas the undifferentiated cytotrophoblasts form the inner layer and give rise to a variety of different trophoblast cells, such as syncytiotrophoblasts [13] or column cytotrophoblasts [19].

**Fig. 1:**
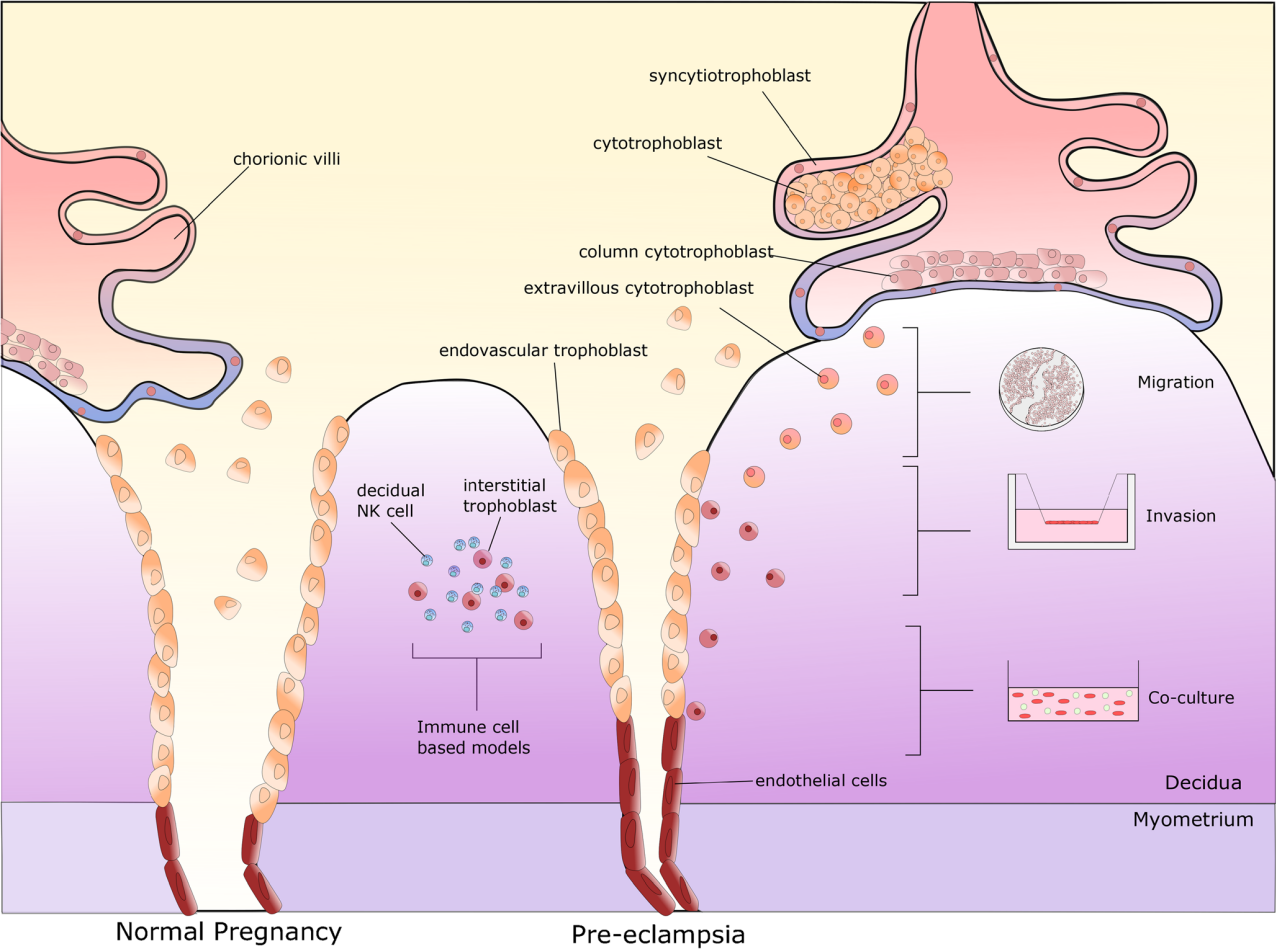
Schematic diagram of spiral uterine artery remodelling by trophoblasts and in vitro assays used to model different stages in this process. Chorionic villi sprouting from the blastocyst consist of two distinct villous trophoblast cell types: syncytiotrophoblasts and cytotrophoblasts. The syncytiotrophoblasts forms the outer layer of the chorionic villi, whereas cytotrophoblast layer is considered stem like. Column trophoblasts are found in the anchoring villi where they form partially complete shell facilitating movement of extravillous trophoblasts through the maternal decidua (migration). Interstitial trophoblasts, upon entering decidua, gather and destroy arterial media (invasion); endothelial cells undergo apoptosis, which allows for their replacement by endovascular trophoblasts (co-culture). The most commonly found lymphocytes in the decidua during pregnancy are natural killer (NK) cells (co-culture)

Syncytiotrophoblasts are in direct contact with maternal blood and thus provide the biochemical barrier between the mother and developing foetus. Within the anchoring villous tips, cytotrophoblasts differentiate into EVTs, which emerge from the cell column and migrate through the maternal decidua with the intention of remodelling the SUA [20, 21]. Endovascular trophoblasts, as their name suggests, enter through the distal lumen of the SUA, whereas interstitial trophoblasts enter via the decidua [22] where they begin the process of remodelling (Fig. 1). Arrival of endovascular trophoblasts leads to the removal of maternal endothelial cells [23]. Invasion of the SUA results in the loss of endothelial lining and elastic tissue. The increase in width of these vessels is partly due to the loss of elasticity, which, in turn, provides a lower resistance vessel enabling blood supply to the developing foetus. Invading trophoblast cells in the SUA replace the original maternal endothelial cells [22].

Despite substantial knowledge in relation to the physiological processes involved in SUA remodelling by trophoblasts, aberrant mechanisms impeding these processes are not well established. Therefore, there is an urgent need for effective experimental models which will help elucidate many of the unknown aspects of inappropriate SUA remodelling leading to pre-eclampsia and aid the development of effective preventive and therapeutic strategies. In this review, we will critically assess existing complementary human and cell line-based in vitro models used to elucidate various mechanisms involved in SUA remodelling, which could be relevant to the pathogenesis of pre-eclampsia.

### Two-Dimensional Cell Migration and Invasion Assays

The invasion assay is a high-throughput method which assesses cellular motility through a permeable membrane therefore representing trophoblast migration through the endometrium.

Within the Rho family of GTPases, Rac1 has been shown to act as a regulator of many important cellular processes, such as migration and invasion [24]. HTR-8/SV.neo trophoblast cells were originally derived from chorionic villi explants and were transfected with the simian virus 40 large T antigen [25]. Fan and colleagues used short hairpin (sh) RNA to silence Rac1 expression in HTR-8/SV.neo cells before performing a transwell Matrigel invasion assay. Following the knockdown of Rac1, HTR-8/SV.neo cells were allowed to grow for an additional 24 h in transwell inserts, before the invading cells were fixed in paraformaldehyde and stained with crystal violet. By counting cells in ten random fields of view, it was shown that Rac1 knockdown significantly reduced migration of HTR-8/SV.neo cells in comparison to the control [26].

Other important regulators of cell migration, elastin-derived peptides (EDPs) have been implicated in the conversion of the SUA into a low resistance vessel [27]. Using this knowledge, Desforges et al. modelled functional aspects of SUA by exposing trophoblast cells, SGHPL-4, to an elastin-derived matrikine, VGVAPG. SGHPL-4 cells are EVTs derived from the first trimester of pregnancy. The invasion of SGHPL-4 cells through a transwell plate was increased when exposed to VGVAPG for 24 h [28].

MicroRNAs are small non-coding RNA molecules that regulate gene expression, through silencing or post-transcriptional regulation therefore affecting stability and translation of messenger RNA (mRNA). Tamaru et al. demonstrated that overexpression of miR-135b significantly reduced the invasive capacity of HTR-8/SV.neo cells, by causing a decrease in the mRNA expression of *CXCL12* by approximately 50%, in low-oxygen conditions [29]. The *CXCL12* gene plays a role in placentation [30] and is linked to the development of pre-eclampsia [31], whereas miR-135b is expressed in trophoblast cells [29]. The functional role of miR-93 has yet to be elucidated in pre-eclampsia; however, the levels of this microRNA are increased within the plasma of patients who developed pre-eclampsia. SOLiD sequencing revealed a total of 20 downregulated microRNAs in plasma samples obtained from five patients, of which four had developed pre-eclampsia [32•]. Furthermore, Pan et al. have shown that miR-93 inhibitors can stimulate trophoblast migration and invasion [33]. Choriocarcinoma is a rapidly growing cancer of the placenta, in particular the chorion. Placental choriocarcinoma-derived cell lines, BeWo and JAR cells, in a transwell chamber also displayed reduced motility when transfected with miR-93 mimetics [33].

As mentioned above, hCG-H is secreted by syncytiotrophoblasts during early placentation [34]; however, it is also secreted by choriocarcinoma cells. Using xCelligence (ACEA, San Diego) real-time cell analysis system, Evans et al. performed migration and invasion assays using JEG-3 cells. Cell invasion was inhibited following a reduction of hCG-H by hCG-H neutralising antibody, whereas there was no effect on cell migration [18].

Metastasis-associated protein-3 (MTA-3) can also regulate cell migration. In pre-eclampsia, the levels of MTA-3 appear to be reduced [35]. Therefore, when Horii et al. generated MTA-3 stable knockdown in choriocarcinoma JEG-3 cells using shRNA, there was a 60% decrease in hCG secretion in the knockdown models compared to control, and migration was increased by 1.8-fold [36].

Moreover, Liu and colleagues have implicated that ephrin-B2 could play an important role in the remodelling of SUA due to its influence on trophoblast cell functionality [37••]. Ephrin-B2 is a transmembrane ligand of Eph receptors, and it belongs to the largest family of receptor tyrosine kinases [38]. It regulates embryonic vascular development and postnatal angiogenesis [39]. Ephrin-B2 and its role in SUA remodelling were analysed using a number of functional assays with HTR-8/SV.neo cells. The migration, invasion and tube formation of HTR-8/SV.neo cells were diminished when ephrin-B2 was knocked down using sh-ephrin-B2. The expression of MMP-2 and MMP-9, key proteins involved in the breakdown of the extracellular matrix and remodelling, was also decreased in the transfected cells [40].

The Notch pathway, a regulator of ephrin-B2 expression [37••], is one of the key angiogenic and stem cell pathways [41]. It is a canonical pathway where ligands such as delta-like (DLL) 1, 3 and 4 on one cell activate notch receptors (1–4) on the neighbouring cells. Inhibition of DLL4 has been shown to promote endothelial cell proliferation, but it leads to irregular pro-angiogenic phenotype, and therefore, it is likely to be implicated in endothelial dysfunction [42]. Protein or RNA expressions of DLL4 and other members of the Notch pathways such as Notch-2, Notch-3, DLL3, JAG1, JAG2, Hey-1 and Hey-2 were all downregulated in cell lysates from placental samples collected from women with pre-eclampsia compared to healthy control placentae [43]. The shRNA downregulation of Notch-2 receptor led to a decrease in BeWo cell migration and invasion, whereas overexpression of Notch-2 led to an increase in the migration and invasion of JAR cells [44]. Conversely to Notch, other stem cell markers, CD44 and CD34, have shown higher expression in placental samples collected from women with pre-eclampsia (*n* = 21) vs. normotensive controls (*n* = 20) [45].

Endothelial progenitor cells (EPCs) are essential in vascular remodelling and endothelial homeostasis [46]. EPCs are able to form new blood vessels, and therefore have a key role in vascular repair [47]. Blood samples from 13 women with pre-eclampsia demonstrated a lower number of EPCs compared to healthy controls. A reduction in EPC number was demonstrated before pre-eclampsia developed clinically [48]. Similarly, a reduced number of endothelial colony-forming cells (ECFCs), which are a subclass of EPCs committed to become endothelial cells [49], was also observed within umbilical cord blood from women with pre-eclampsia [50••], suggesting that the reduction in EPCs is present both prior and after pre-eclampsia develops. The reduction and dysfunction of EPCs reflects the lack of endothelial repair capacity in pre-eclampsia [51]. Liu and colleagues isolated and cultured EPCs from umbilical cord blood and placentae from 12 women with pre-eclampsia and 12 healthy pregnant women at delivery [37••]. Western blotting and RT-PCR results using isolated EPCs demonstrated higher ephrin-B2 mRNA and protein levels in women with pre-eclampsia. This was also true within placental samples. Furthermore, the number of EPCs isolated from umbilical cord were negatively correlated with the expression of ephrin-B2 levels in placentae. A small molecule-based activation of DLL4 and Notch pathway led to activation of ephrin-B2 in EPCs and inhibition of EPC activity [37••]. In conclusion, Notch or ephrin-B2 could be potential targets capable of repairing angiogenesis in patients with pre-eclampsia [37••].

Two-dimensional cell culture assays, such as the migration or invasion assay, provide a useful tool to study the functionality of trophoblast cells. Despite their importance, it is necessary to conduct further three-dimensional assays to better mimic human environment.

### Three-Dimensional Cell Culture-Based Models

Time-lapse microscopy, three-dimensional (3D) invasion and tube formation assays were all used by Wallace et al. to assess trophoblast functionality in the presence of increasing concentrations of angiogenin or endostatin. Invasion and tube formation were reduced significantly in the presence of endostatin, whereas angiogenin decreased invasion but increased tube formation. Similarly, when fibrin gel assay was used to determine the volume of invading trophoblast cells from a 3D spheroid (stimulated by endothelial growth factor, and in the presence of endostatin), the invasive ability of SGHPL-4 trophoblast cells was decreased considerably. On the other hand, there was no change in cell motility by endostatin or angiogenin [52•].

A 3D co-culture model of trophoblast cells and endometrial adenocarcinoma cells demonstrated interesting results in relation to invasive capacity of trophoblasts in this system. Three different endometrial adenocarcinoma cell lines were used to emulate epithelial cells of the endometrium: the HEC-1-A cell line, the RL95-2 cell line and the Ishikawa cell line. Endometrial epithelial cells (EECs) were mixed with Matrigel, and once solidified, medium containing the same cell line was added to the chamber slide. Following 4-day incubation, the EECs formed spheroids; trophoblast cells (AC-1M88) were, then, added to EEC spheroids. Trophoblast cells attached to the EECs forming a cell monolayer. Out of three endometrial cell lines, trophoblast cells were able to invade RL9-2 cells the most effectively. Interestingly, the RL9-2 spheroids formed in the Matrigel showed the least polarisation out of the three endometrial cell lines. A higher extent of differentiation and polarisation decreased trophoblast invasion [53••].

In co-culture experiments carried out by Virtanen and colleagues, human CRL-2522 fibroblasts and HUVEC displayed a pro-angiogenic phenotype. Similarly, a co-culture of human adipose stem cells (hASCs) and HUVEC demonstrated activation of angiogenesis and vasculogenesis. However, when cord blood serum from women with pre-eclampsia was added to these co-culture models, tubule formation was inhibited in both models compared to when serum from normotensive cord blood was added; this reflects the anti-angiogenic state that is present in pre-eclampsia [54].

Women with gestational diabetes mellitus (GDM) have increased risk of developing pre-eclampsia, up to 10% [55, 56]. Therefore when feto-placental endothelial cells were cultured with conditioned media derived from trophoblasts isolated from patients with GDM, the cells migrated slower in a wound scrape assay and showed reduced chemo-attraction/migration through chamber pores [57]. Furthermore, when Loegl et al. investigated feto-placental angiogenesis in women with GDM, primary trophoblast-conditioned media from women with GDM led to increased tube formation but reduced wound healing and chemo-attraction ability [57]. GDM also altered expression and secretion of pro-angiogenic and anti-angiogenic factors therefore leading to changes in placental angiogenesis and vascular structure, which is common in GDM pregnancies [57]. Other nutrient-sensing pathways relevant to DM, such as the AMPK pathway the main target of metformin, have been implicated in trophoblast functionality and endothelial function, and hence, it could be a relevant target for prevention of pre-eclampsia [58–60].

### Cell Survival and Proliferation

As previously explained, the cytotrophoblast cells undergo proliferation during hypoxic conditions; survival and proliferation are essential for appropriate and efficient remodelling of SUA. Following invasion of the SUA lumen, EVTs must survive long enough to carry out remodelling functions.

Pre-implantation factor (PIF) is a peptide secreted by embryos, which has been implicated in trophoblast invasion of SUA [61, 62]. Placentae collected from women with pre-eclampsia demonstrated lower placental protein levels of PIF compared to healthy controls [61]. Moindjie and colleagues used a synthetic PIF analogue (sPIF) to elucidate its role in early stage trophoblast apoptosis. Using an annexin V-FITC staining assay by flow cytometry, the number of apoptotic HTR-8/SV.neo cells was reduced in the presence of 50 or 100 nM sPIF by 26.3 and 39.6%, respectively [63]. Furthermore, in a late-stage apoptosis (DNA fragmentation) assay, sPIF treatment showed a significantly lower apoptotic index when assessed by a terminal deoxynucleotidyl transferase-mediated dUTP-biotin DNA-nick end labelling (TUNEL) assay [63].

In relation to ephrin-B and Notch signalling, in addition to their role in migration and invasion, the knockdown of ephrin-B2 showed a small reduction in cell proliferation, assessed by the CCK-8 assay. Similarly, a higher number of apoptotic cells was observed via Hoechst 33258 staining following a knockdown of ephrin-B2 [40]. In relation to Notch receptors, proliferation of JAR cells was decreased when Notch-2 was overexpressed; however, the knockdown of Notch-3 led to an increase in proliferation. Notch-2 knockdown increased the volume of BeWo cells undergoing the S phase of the cell cycle, effectively increasing proliferation [44].

Matricellular proteins of the CCN family also play a role in trophoblast proliferation and migration [64]. Within this family of proteins, CCN1 and CCN3 are known to affect cell growth, as well as cell migration [65]. Following treatment with recombinant CCN1 and CCN3 proliferation of SGHPL-5 trophoblast cells was reduced and the cell cycle progression arrested [65].

In summary, 3D cell culture models have an advantage over 2D models because these resemble the human environment better, enabling assessment of direct and indirect cell-cell interactions as well as interactions between the cells and the surrounding environment. Overall, 3D models are more effectively used with primary cells and tissue explants than with cell lines. Summary of the molecular mechanisms identified using 2D and 3D in vitro models are presented in Table 1.

**Table 1:** Summary table of the molecular mechanisms implicated in trophoblast or placental functionality

Cell or tissue type	Stimulus	Effect	Ref.
HTR-8/SV.neo	Rac1 shRNA	Reduced migration Snail expression was considerably reduced	[26]
SGHPL-4 cells	Elastin-derived matrikine, VGVAPG	Increased invasion	[28]
HTR-8/SV.neo cells	Overexpression of miR-135b	Reduced invasion *CXCL12* mRNA expression downregulated	[29]
Patient plasma (pre-eclampsia vs. healthy)	SOLiD sequencing	Twenty microRNAs downregulated	[32•]
BeWo and JAR cells	miR-93 inhibitors	Reduced invasion	[33]
Feto-placental endothelial cells derived from the third trimester	Conditioned media derived from trophoblasts isolated from patients with gestational diabetes mellitus (GDM)	Reduced migration Increased tube formation	[57]
JEG-3 cells	hCG-H neutralising antibody	reduced invasion, no effect on migration	[18]
JEG-3 cells	shRNA MTA-3 stable knockdown	hCG secretion reduced Migration increased	[36]
HTR-8/SV.neo cells	Ephrin-B2 knocked down using sh-ephrin-B2	Migration, invasion and tube formation diminished MMP-2 and MMP-9 expression decreased Cell proliferation reduction	[40]
Cell lysates, from placental samples (pre-eclampsia vs. healthy)	Protein and RNA isolation	DLL3/DLL4/Notch-2/Notch-3/JAG-1/JAG-2/Hey-1/Hey-2 downregulated	[43]
BeWo	shRNA downregulation of Notch-2 receptor	Reduced migration and invasion Increased proliferation	[44]
JAR	Overexpression of Notch-2	Increased migration and invasion Decreased proliferation	[44]
JAR	Knockdown of Notch-3	Increased proliferation	[44]
Placental samples (pre-eclampsia vs. healthy)	ELISA/flow cytometry	Higher expression of CD44 and CD34	[45]
Blood samples (pre-eclampsia vs. healthy)	Isolation of EPCs from peripheral blood	EPCs considerably lower	[48]
Cord blood (pre-eclampsia vs. healthy)	Isolation of ECFCs	Reduced number of ECFCs	[50••]
EPCs from umbilical cord blood and placentae (pre-eclampsia vs. healthy)	Western blotting and RT-PCR	Higher ephrin-B2 mRNA/protein levels EPCs negatively correlated with the expression of ephrin-B2	[37••]
SGHPL-4	Angiogenin or endostatin	Reduced invasion and tube formation with endostatin Decreased invasion and increased tube formation with angiogenin No change in cell motility	[52•]
AC-1 M88 cells	3D co-culture model	A higher extent of differentiation and polarisation decreased trophoblast invasion Trophoblast cells were able to invade RL9-2 cells the most effectively	[53••]
Maternal and umbilical cord blood samples	Cord blood serum	CRL-2522 fibroblasts and HUVEC co-cultured, displayed a pro-angiogenic effect hASC and HUVEC co-cultured, promoted angiogenesis/vasculogenesis Cord blood serum from women with pre-eclampsia, reduced tubule formation	[54]
Primary trophoblasts (pre-eclampsia vs. healthy)	Plasma pre-implantation factor (PIF)	Increased invasion	[61]
HTR-8/SV.neo	Synthetic PIF analogue (sPIF)	Apoptosis reduced	[63]
SGHPL-5	Recombinant CCN1 and CCN3	Proliferation reduced Cell cycle progression arrested	[65]
Placental explants	EDP mimetic, VGVAPG	Outgrowth area and migration distance significantly larger	[28]
First trimester chorionic villi	Endothelin-1	Trophoblast outgrowth was decreased Reduced invasion	[67]
Placental tissues (pre-eclampsia vs. healthy)	Western blotting/RT-PCR/IHC IHC	Mapsin mRNA/protein levels higher IHC: increased and more diffused staining of mapsin	[70]
Placental tissue (pre-eclampsia vs. healthy)	IHC	IHC: more intense staining of calcyclin in the trophoblasts isolated from patients with pre-eclampsia	[73]
HTR-8/SV.neo	Leukocytes pre-treated with FTY720, an S1P analogue	Reduced migration	[96]
HUVEC	NK cells treated with FTY720, an S1P analogue	Reduced HUVEC tubule formation	[96]
Placental CD74-positive macrophages	CD74 was silenced by siRNA	Reduced ability to adhere to trophoblast cells	[98]
SGHEC-7	dNK-conditioned media (impaired SUA remodelling vs. healthy)	Reduced endothelial cell destabilisation	[103••]

### In Vitro Models with Placental Explants or Primary Trophoblast Cells

The role of EDPs has already been discussed above in relation to their stimulatory effect on migration and invasion using cell lines. Here, the effect of EDPs was also investigated using placental explants. Following the removal of villous tips from the first trimester placental tissue, extravillous trophoblast outgrowths were derived and placed onto collagen. These explants were allowed to adhere, before media containing the EDP mimetic, VGVAPG, was added. Villi were imaged at several time points; at each point, the area covered and the distance of growth travelled were quantified. Outgrowth area and migration distance in the presence of the EDP were shown to be significantly larger than that of the control [28].

Key proteins involved in tissue remodelling, MMPs, particularly MMP-14 and MMP-15, appear to also play an important role in SUA remodelling [66, 67]. When first-trimester chorionic villi were cultured in the presence of endothelin-1, a potent vasoconstrictor upregulated in pre-eclampsia [68], trophoblast outgrowth was decreased by 24% [67]. Invasion was also reduced by 26% following treatment with 100 nM of endothelin-1. The mechanism implicated in the inhibitory effect of endothelin-1 was linked to downregulation of MMP-14 and MMP-15 [67].

Mapsin is an epithelial-specific class II tumour suppressor gene, which has been shown to have inhibitory actions on the invasion of breast cancer cells. In addition to this, mapsin’s role in placental development and invasion of cytotrophoblasts has also been demonstrated [69]. In Liu et al., placental tissues were collected after delivery from 12 women with pre-eclampsia and 12 healthy controls. Western blotting, RT-PCR and immunohistochemistry (IHC) were conducted to assess mapsin expression in placentae from women with pre-eclampsia and matched group of healthy controls. The degree of methylation in the promoter regions of mapsin in each of the study groups was assessed. The patients were matched in terms of age, body mass index (BMI), gestational age and parity. They found mapsin mRNA and protein levels to be significantly higher in pre-eclampsia group compared to healthy controls. IHC of placental tissue showed an increased and more diffused staining of mapsin in pre-eclampsia [70].

Moreover, calcyclin or S100A6 protein, a Ca^2+^channel-binding protein which belongs to the S100 family of proteins, is upregulated with the cellular stress response [71]. It is differentially expressed in trophoblast cells isolated from pregnancies complicated by pre-eclampsia compared to healthy controls [72]. Schol et al. investigated biomarker potential of calcyclin in formalin-fixed and paraffin-embedded placental tissue collected from 75 women with pre-eclampsia and the same number of healthy controls who delivered between 20 and 34 weeks of gestation. IHC analysis showed a significantly more intense staining of calcyclin in the trophoblasts from women with pre-eclampsia compared to healthy controls [73]. Determining expression of trophoblasts’ calcyclin early in pregnancy could be useful to investigate its role in the pathogenesis of pre-eclampsia. However, obtaining placental samples early in pregnancy through chorionic villus sampling is associated with a high incidence of miscarriage; if calcyclin’s role in the pathogenesis of pre-eclampsia is to be further validated, the most convenient method to measure its levels early in pregnancy would be by using peripheral blood or urine sample. Whether these samples correspond to the placental levels needs to be investigated further.

Other important proteins and potential markers of pre-eclampsia identified by IHC of the placental tissues are included in Table 2 [61, 70, 74••, 75–87].

**Table 2:** Important cell markers within placental tissue and their relevance in pre-eclampsia

Protein		Target cells	Relevance	Origin of placenta (trimester)			Ref.
				I	II	III	
α-Smooth muscle actin		Vascular smooth muscle cells of SUA	SUA remodelling			+	[74••]
		Decidual mesenchymal stem cells (DMSCs)				+	
Angiogenin		Syncytiotrophoblasts Cytotrophoblasts	Important for placental vasculogenesis and organogenesis		+		[75]
CRIPTO-1		EVT	Increased in creta placentae			+	[76]
Cytokeratins	AE1/AE3	CVTs EVTs	Epithelial differentiation and pan-trophoblast marker	+		+	[61]
	CK5	CVTs EVTs	Epithelial differentiation marker			+	[77, 78]
	CK7/CK18/CK19	CVTs EVTs	Epithelial differentiation marker Decreased significantly in pre-eclampsia			+	[77-79]
	CK8	CVTs EVTs Amniotic epithelium	Epithelial differentiation marker Decreased significantly in pre-eclampsia			+	[77, 78]
	CK14	BeWo cells Primary trophoblast cells Amniotic epithelium	Epithelial differentiation marker Trophoblast progenitor cells markers			+	[77, 79]
	CK20	CVTs EVTs	Marker of molar pregnancy			+	[77]
E-Cadherin		Syncytiotrophoblasts	Epithelial differentiation marker Increased in pre-eclampsia			+	[77, 79]
EGF receptor splice variant (p110/EGFR)		Syncytiotrophoblasts	EGF receptor antagonist Increased in pre-eclampsia			+	[80]
Epidermal growth factor (EGF)		Villous cytotrophoblasts	Decreased in pre-eclampsia			+	
Endoglin (Eng) or CD105		Syncytiotrophoblasts Endothelial cells	Increased in pre-eclampsia			+	[81]
Galectin-2		Syncytiotrophoblasts	Decreased in pre-eclampsia			+	[82]
HIF-1α		Syncytiotrophoblasts	Increased in pre-eclampsia			+	[83]
HLA-G		CVTs EVTs	Identification of cytotrophoblasts and EVTs	+		+	[61]
Maspine		CVT Endothelial cells	Increased and more diffused expression in pre-eclampsia			+	[70]
Matrix metalloproteinase type 9		Decidual cells	Increased in pre-eclampsia			+	[84]
Matrix metalloproteinase types 1 and 3		Decidual cells Interstitial EVTs	Important for trophoblast invasion and remodelling of SUA Increased in pre-eclampsia			+	[84]
Placental growth factor (PlGF)		Syncytiotrophoblasts	Decreased in pre-eclampsia			+	[83]
Pre-implantation factor (PIF)		EVT CVT	Important for trophoblast invasion and placentation	+		+	[61]
pSTAT3		Endothelial cells of SUA	Increased in pre-eclampsia			+	[85]
Pyruvate kinase M2 (PKM2)		Syncytiotrophoblasts	Increased expression in pre-eclampsia			+	[86]
VEGF	R1	Trophoblasts Immune cells	Increased expression in pre-eclampsia (83)/no change (85)			+	[85, 87]
	R2	Endothelial cells	No change in pre-eclampsia			+	[85]
	R3	Trophoblasts Endothelial cells	Expression decreased in endothelial cells in pre-eclampsia			+	[85]
Vimentin		Stromal mesenchymal cells of chorionic villi	Cell identification			+	[77]

SUA, spiral uterine artery; PMC, placental mesenchymal cells; CVT, chorionic villous trophoblasts; EVT, extravillous trophoblasts; dNK, decidual natural killer

In addition to trophoblasts, other groups of cells, which are important for appropriate SUA remodelling and placental development, include immune cells.

### In Vitro Immune Cell-Based Models

Adequate signalling between foetal and maternal immune cells is an essential requisite to achieve adequate and early pregnancy placentation, vasculogenesis and immune tolerance of the foetus [88]. Natural killer (NK) cells are a major source of angiogenic growth factors and cytokines that ensure the transformation of the SUA, foetal implantation and placentation [89–91]. Abnormal NK cell receptors and cytokine production profile are associated with pregnancy disorders, such as pre-eclampsia [92]. Interferon-gamma secretion by NK cells is an essential regulator of vascular remodelling and EVT migration. Lowered levels of interferon-gamma were observed in decidual NK (dNK) cells from pregnant women with hypertensive disorders [93].

Major histocompatibility complex class I-related chain (MIC) genes are stress-inducible proteins modulating the function of immune NK cells. Engagement of NKG2D receptor by MIC genes has been shown to stimulate NK cell-mediated cytokine production, and the release of soluble MIC proteins was also suggested to modulate NK cell function during pregnancy [94, 95].

Sphingosine-1-phosphate (S1P) has been shown to regulate numerous functions of NK cells therefore having a potential role in SUA remodelling. Therefore, when Zhang et al. cultured HTR-8/SV.neo cells overnight in serum-free media, before wounding a confluent monolayer of cells and adding leukocytes pre-treated with FTY720, an S1P analogue, the migration was considerably reduced [96]. The extent of migration was analysed by photographing 10 randomly chosen areas of the wound scrape. This way of assessing migration could lead to inconsistencies due to moderate variations in the wounding process. Wound sizes can be variable, and, as a result, this only provides a rough basis for migration with reduced reproducibility. The use of culture inserts with a defined free cell gap can provide a standardised wound size, avoiding any cell damage. Using live cell microscopy imaging with a Mark & Find/Tile Scan feature allows for identical sections of a well to be imaged, with the use of pre-defined co-ordinates.

When primary trophoblast cells from villous tips were added to Matrigel-coated inserts, in the presence of NK cells pre-treated with FTY720, a reduction in trophoblast migration was observed. When NK cells pre-treated with FTY720 were cultured with HUVEC, inhibition of tubule formation was also observed. These results stipulate an important role of NK cells and S1P in the process of SUA remodelling and developmental angiogenesis [96].

In the study by Haumonte et al., peripheral blood was collected from a cohort of 81 pregnant women diagnosed with vascular pregnancy diseases including 40 women with pre-eclampsia and 63 healthy pregnancies matched for age, BMI and parity. Plasma levels of soluble MIC (sMIC), NKG2D and interferon-gamma were measured using ELISA, flow cytometry and RT-PCR. Plasma samples, which were positive for sMIC, demonstrated higher incidence of pre-eclampsia and proteinuria and lower levels of NKG2D and interferon-gamma. Therefore, the presence of sMIC molecules in maternal plasma may play a critical role in altering maternal immune functions which is essential for appropriate vascular remodelling during pregnancy [97].

Furthermore, placental growth is facilitated by the interaction between trophoblast and immune cells [98]. The importance of the immune system is further confirmed by the fact that inflammatory markers are raised in pre-eclampsia [99]. Hofbauer cells are placental macrophages of foetal origin and play a direct role in early placental development [98]. These cells are associated with several pregnancy complications, such as chorioamnionitis, spontaneous abortion and foetal metabolic storage disease. They contribute to the placental expression of anti-angiogenic factors, and they appeared to be aberrant in placentae from women with pre-eclampsia [100]. The human leukocyte antigen class II histocompatibility antigen-gamma chain, also known as cluster of differentiation 74 (CD74), when expressed on cell surfaces, is the major histocompatibility complex (MHC) II invariant chain protein that is involved in antigen presentation and crucial for biogenesis [101]. CD74 has also a high affinity binding protein for the pleiotropic inflammatory cytokine macrophage migration inhibitory factor (MIF). Przbyl et al. hypothesised that CD74 has an important role in aberrant placentation in pre-eclampsia. Indeed, the number of CD74-positive macrophages was significantly lower in placental samples collected from women with pre-eclampsia (*n* = 50) compared to healthy controls (*n* = 28) [98]. When CD74 was silenced by siRNA, macrophages displayed a reduced ability to adhere to trophoblast cells in comparison to the control. The Ingenuity Pathway Analysis Tool determined that the gene expression levels of activated leukocyte cell adhesion molecule (ALCAM), intracellular adhesion molecule 4 (ICAM4) and Syndecan-2 (SDC-2), known to be involved in cell adhesion, were considerably reduced [98].

Within the decidua during early pregnancy, NK cell population is abundant, making up 50–90% of the total lymphocyte count [102]. Although dNK cells are present during SUA remodelling, their function has not yet been fully elucidated in relation to endothelial remodelling and integrity. Fraser et al. attempted to address this question by isolating dNK cells at 9–14 weeks of gestation from healthy pregnancies and pregnancies that showed abnormal uterine artery Doppler results as per resistance indices. The decidua was processed, and CD56^+^ cells were sorted and cultured as dNK cells for 24 h before conditioned growth media was removed and pooled together. SV40 transfected human umbilical vein endothelial cells (SGHEC-7) were plated in Angiogenesis ibiTreat chamber slides containing Matrigel and allowed to form tube-like structures. Standard culture media were then replaced with dNK-conditioned media. Prior to invasion of SUA by trophoblasts, the SUA must undergo various physiological changes in their structure including forming gaps in the endothelial layer to promote invasion (Fig. 1). SGHEC-7 cells cultured with dNK-conditioned media from pregnancies with abnormal Doppler results did not show endothelial cell destabilisation to the same extent as those cultured in dNK-conditioned media from healthy pregnancies. This implies that dNK have a role in disrupting endothelial structure and that a reduction in this disruption may be a contributing factor to the inadequate SUA remodelling. The likely mechanism of this effect is not apoptotic but rather pro-inflammatory involving TNF-α signalling [103••].

Similar to Fraser et al., another group isolated dNK cells from healthy pregnancies and pregnancies with abnormal uterine artery Doppler or high-resistance index, with an aim of profiling cytokines and angiogenic factors secreted in the culture media of dNK cells. Both angiogenin and endostatin were produced at a higher level by dNK cells from pregnancies with high resistance index. Endostatin inhibited trophoblast invasion and endothelial-like trophoblast tube formation, while angiogenin inhibited trophoblast invasion but promoted tube formation. In summary, altered expression of angiogenin and endostatin which are secreted by dNK cells may contribute to pregnancy complication associated with SUA remodelling [52•].

## Conclusion

The current available in vitro systems used to model pre-eclampsia have so far helped to elucidate numerous key proteins potentially involved in the development of this condition (Tables 1 and 2). However, there are still many aspects of the pathogenesis of pre-eclampsia that remain unknown. Although cell culture models remain essential to study the mechanisms of diseases, pre-eclampsia is a complex and multifactorial disease of unknown aetiology, and, as such, it is important to appreciate the limitations of these models. Selecting appropriate assays to elucidate key features of the pathophysiology of pre-eclampsia is necessary to ensure the generation of robust results. Currently, there is notable variation between experimental designs of the same assays, so perhaps standardisation of the methodology may attempt to address this, leading to greater reproducibility. Co-culture and 3D in vitro models, particularly with primary cells, help to mimic a more realistic cellular environment and provide preliminary data important for the development of future or repurposed therapies. Nevertheless, propagation of cells and manipulation of expression of various key proteins are only possible with cell lines. Furthermore, in vivo animal models have the advantage of pre-clinical testing of a therapeutic; however, initial in vitro work generally precedes in vivo experimental phase. Even though ex vivo assays only partly emulate an in vivo setting, they allow us to underpin key molecules or pathways in a disease.

In summary, despite a substantial progress which has been made in the field of pre-eclampsia and in relation to understanding the function and key signalling mechanisms of trophoblast cells and SUA remodelling, further studies are required to fully elucidate the mechanisms involved in the pathogenesis of pre-eclampsia. Identification of the key pathways involved in placental dysfunction, which is the root cause of pre-eclampsia, can lead to the development of reliable and early biomarkers of pre-eclampsia and effective preventative treatment strategies.
